# Visualisation of ribosomes in *Drosophila* axons using Ribo-BiFC

**DOI:** 10.1242/bio.047233

**Published:** 2020-01-02

**Authors:** Anand K. Singh, Akilu Abdullahi, Matthias Soller, Alexandre David, Saverio Brogna

**Affiliations:** 1 School of Biosciences, University of Birmingham, Edgbaston, Birmingham B15 2TT, UK; 2 Oncology Department, Institut de Génomique Fonctionnelle, 141 rue de la Cardonille, 34094 Montpellier cedex 5, France

**Keywords:** BiFC, 80S, Ribosomes, Neurons, Axons, *Drosophila*

## Abstract

The distribution of assembled, and potentially translating, ribosomes within cells can be visualised in *Drosophila* by using Bimolecular Fluorescence Complementation (BiFC) to monitor the interaction between tagged pairs of 40S and 60S ribosomal proteins (RPs) that are close neighbours across inter-subunit junctions in the assembled 80S ribosome. Here we describe transgenes expressing two novel RP pairs tagged with Venus-based BiFC fragments that considerably increase the sensitivity of this technique we termed Ribo-BiFC. This improved method should provide a convenient way of monitoring the local distribution of ribosomes in most *Drosophila* cells and we suggest that it could be implemented in other organisms. We visualised 80S ribosomes in different neurons, particularly photoreceptors in the larva, pupa and adult brain. Assembled ribosomes are most abundant in the various neuronal cell bodies, but they are also present along the full length of axons. They are concentrated in growth cones of developing photoreceptors and are apparent at the terminals of mature larval photoreceptors targeting the larval optical neuropil. Surprisingly, there is relatively less puromycin incorporation in the distal portion of axons in the larval optic stalk, suggesting that some of the ribosomes that have initiated translation may not be engaged in elongation in growing axons.

This article has an associated First Person interview with the first author of the paper.

## Abstract (precis)

**Summary:** We described a novel straightforward technique termed Ribo-BiFC to visualise assembled ribosomes in *Drosophila* cells. This technique revealed ribosomes in axons of both developing and mature neurons.

## INTRODUCTION

Ribosomes are ubiquitous molecular machines that translate gene sequences into the thousands of different proteins that make and operate every organism, so ribosomal components are some of the most abundant and evolutionarily conserved macromolecular constituents of cells. Each ribosome is made up of two complex ribonucleoprotein subunits – 40S and 60S in eukaryotes – and the joining of these into 80S functional ribosomes is tightly regulated. Even when cells are replete with ribosome subunits there are physiological situations (e.g. during nutrient deprivation or other cell stresses) when relatively few are assembled into protein-translating ribosomes (Hinnebusch, 2014, 2017).

The joining of ribosomal subunits is a multi-step process, requiring the coordinated activity of several initiation factors, occurring each time that translation of an mRNA is initiated (Hinnebusch, 2017; Jackson et al., 2010). In eukaryotes, the first step is activation of the 40S subunit, which starts with its loading with methionine initiator tRNA (tRNAi^met^). The resulting pre-initiation complex, typically guided by an interaction with the eukaryotic translation initiation factor eIF4G which is bound to the 5′ end cap-associated eIF4E, then attaches to the mRNA and scans its 5′UTR until the initiation codon is recognised by base pairing between the anticodon of tRNAi^met^ and an AUG start codon (Kozak, 1989). Once tRNAi^met^ is base-paired with the AUG and is precisely placed in the peptidyl site on the 40S subunit, the 60S subunit is recruited. The assembled 80S ribosome translocates along the mRNA, catalysing protein synthesis until it reaches a stop codon. It then dissociates and the free subunits become available for new rounds of translation (Dever and Green, 2012).

We have used the Bimolecular Fluorescence Complementation (BiFC) technique to visualise assembled ribosomes in *Drosophila* cells. This is a technique that allows direct detection of diverse types of protein–protein interactions in living cells (Hu et al., 2002; Kerppola, 2008). To apply this for ribosomes, one selects a pair of RPs on the surface of the individual subunits that only come into close and stable contact when the 80S ribosome assembles. These RPs are then tagged with functionally complementary halves of a fluorescent protein. The two non-functional halves of the fluorescent protein only make a stable contact when the 80S ribosome is assembled at initiation, so emission of fluorescence reports that translation initiation has occurred (Al-Jubran et al., 2013).

**Fig. 1.**
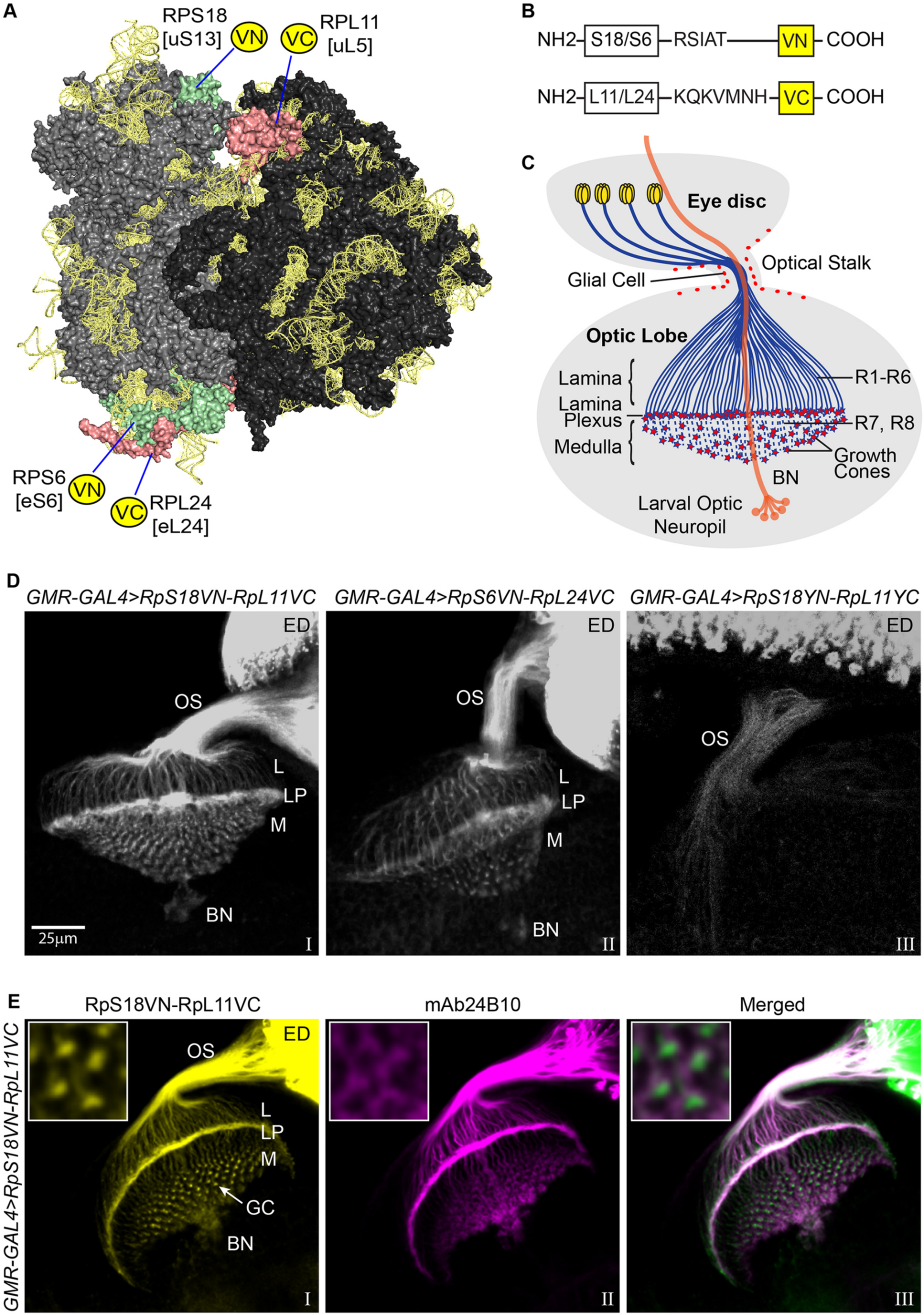
**Ribo-BiFC visualisation of 80S ribosomes in photoreceptors.** (A) Model of the *Drosophila* 80S ribosome with the two BiFC tagged RP pairs on the small and large subunits highlighted: RpS18/RpL11 [uS13/uL5] and RpS6/RpL24 [eS6/eL24]; the image was generated with PyMOL using the published high-resolution *Drosophila* 80S structure, PDB file 4V6W (Anger et al., 2013). RpS18 and RpS6 on the 40S are indicated in pale green, RpL11 and RpL24 on the 60S in pale red. (B) Diagram of the Bimolecular Fluorescence Complementation (BiFC) constructs with spacer sequences indicated, the VN and VC BiFC-compatible fragments of Venus fluorescent protein are shown as yellow boxes. (C) Schematic of the eye disc connected by the optic stalk to the brain optic lobe of *Drosophila* larva, showing the photoreceptor cell bodies in the retina (yellow) and their axonal projections into the brain (blue). The photoreceptors R1-R6 project their axons to the lamina region of the brain, while R7 and R8 project their axons further inside to the medulla underneath. The star shapes (red) at the end of axons indicate growth cones. Bolwig's nerve (BN, orange) passes through the lamina/medulla and innervates the larval optic neuropil in each lobe. (D) Confocal microscopy images showing the BiFC signal produced by different transgene combinations expressed in the developing photoreceptors using *GMR-GAL4>RpS18VN/RpL11VC* (panel I), >*RpS6VN/RpL24VC* (panel II) and as comparison the YFP-based >*RpS18YN/RpL11YC* (panel III). (E) Visualisation of the RpS18VN-RpL11VC (yellow, panel I) in tissues where the developing photoreceptors are immunostained by mAb24B10 (magenta, panel II), their colocalisation is shown in the merged image (panel III); the RpS18VN-RpL11VC BiFC signal is shown in green instead of yellow in the merged image for better contrast. Insets show magnified views of growth cone region. Labels refer to: ED, eye disc; OS, optic stalk; L, lamina; LP, lamina plexus; M, medulla; GC, growth cone; BN, Bolwig's nerve.

Initially, when we were developing the BiFC-based ribosome visualisation technique, several pairs of RPs were tagged with either the N-terminal half (YN) or the C-terminal half (YC) of Yellow Fluorescent Protein (YFP). These were co-expressed in *Drosophila* S2 cells and only those pairs that come together when the 80S ribosome assembles gave rise to ribosomal fluorescence (Al-Jubran et al., 2013). Moreover, the fluorescence was enhanced by translation elongation inhibitors that stabilise the 80S, and was reduced by initiation inhibitors (Al-Jubran et al., 2013). We then designed transgenic flies encoding one such adjacent pair of RPs under UAS regulation (RpS18[uS13]-YN and RpL11[uL5]-YC) – the names in brackets follow a newer system of naming ribosomal proteins, the prefix ‘u’ (for universal) indicates the protein is conserved in all domains of life (Ban et al., 2014). Here we used the *Drosophila* nomenclature of our previous study to avoid confusion; however, both names are given when a protein is first mentioned in the text or in Fig. 1. When these were expressed in salivary glands, a translationally very active tissue that secretes copious amounts of glue proteins (Andrew et al., 2000; Beckendorf and Kafatos, 1976), the tissue showed an intense 80S ribosomal fluorescence signal (Al-Jubran et al., 2013).

We investigated whether a similar approach could track ribosomes in axons and synapses, and hence serve as a tool for studies of localised translation in the *Drosophila* nervous system (Glock et al., 2017; Holt et al., 2019; Kim and Jung, 2015). Using the available transgenic flies expressing RpS18-YN and RpL11-YC, however, we were only able to detect weak 80S ribosomal fluorescence in the cell bodies of some large neurons. So we sought to improve the sensitivity of this technique we termed Ribo-BiFC. Here we describe an improved version that employs transgenic flies expressing either of two novel RP pairs (RpS18/RpL11 and RpS6[eS6]/RpL24[eL24]) – the prefix ‘e’ is for eukaryotic ribosomal proteins without bacterial homologs – that are tagged with BiFC fragments of Venus fluorescent protein (Hudry et al., 2011). These Venus-based reporters greatly improved the sensitivity of the method and revealed clear ribosome signals along the full length of axons and at the axon terminals of both developing and mature neurons. In eye photoreceptor axons, which we examined in most detail, intense ribosome signals are particularly apparent in their growth cones during larval and pupal development. We suggest that these Venus-tagged RP pairs can provide a useful research tool with which to monitor the subcellular localisation and trafficking of assembled ribosomes in most *Drosophila* cells and tissues.

## RESULTS

### BiFC-Venus-tagged 80S ribosomes can be detected in axons and growth cones of photoreceptor neurons

The ribosomal protein pairs RpS18/RpL11 (uS13/uL5) and RpS6/RpL24 (eS6/eL24) span inter-subunit potential contact points, on the surface of the ‘head’ and the ‘foot’, respectively, of the 80S ribosome (Fig. 1A). We generated UAS-driven *Drosophila* transgenes encoding these proteins that were tagged with complementing fragments of Venus fluorescent protein corresponding to the N-terminal domain (VN, 1-173 aa) and C-terminal domain (VC, 155-238 aa) (Fig. 1B). These yield a brighter and more specific BiFC interaction than YFP constructs (Hudry et al., 2011). Moreover, our characterisation in S2 cells indicated that fluorescence from the inter-subunit Venus BiFC complex might be more stable during translation elongation than the one from the corresponding YFP complex (Al-Jubran et al., 2013).

We tested the new transgenes in the *Drosophila* larval visual system, which is an excellent model for microscopic visualisation of the axonal projections of neurons. The eye is made up of about 750 ommatidia, each having eight photoreceptor neurons (the R-cells: R1-R8). R1–R6 axons project to a synaptic layer of the brain optic lobe termed the lamina plexus, and R7 and R8 axons pass through the lamina and end in a deeper brain region termed the medulla (Fig. 1C) (Mencarelli and Pichaud, 2015). Expression of either of our BiFC-Venus RP pairs in developing eye by using the *GMR-GAL4* driver (Freeman, 1996) results in a strong signal. Within the growing photoreceptors, this is brightest in the cell bodies located in the developing eye, but it is apparent along the entire length of the photoreceptor axons, both in R1-R6 (ending in the lamina) and in R7 and R8 (ending in the medulla) (Fig. 1D; panel I, RpS18/RpL11; Panel II, RpS6/RpL24). The RpS18/RpL11 pair was used in the experiments described below.

The signal from the Venus-based reporters is much stronger than from the previous YFP-based RpS18/RpL11 transgene pair, which was only apparent in the cell bodies and proximal regions of the axons (Fig. 1D, panel III). This was despite the fact that substantial amounts of conventional GFP- or RFP-tagged versions of RpS18 and RpL11, which report the distributions of free ribosomal subunits as well as assembled ribosomes, are abundantly present throughout the axons when expressed with *GMR-GAL4* (Fig. S1A). Although the expression levels of the tagged proteins could not be directly assessed in photoreceptors, as these make up only a small fraction of the cells in the tissue, our previous western blotting analysis of salivary glands indicates that these tagged proteins are at a substantially lower level than the endogenous counterparts, even when expressed in salivary glands with a strong *GAL4* driver that results in a BiFC signal much brighter than that detected in the photoreceptors (Al-Jubran et al., 2013). Moreover, there is no evidence of proteins being considerably toxic when expressed with *GMR-GAL4* since the eye develops as expected, except for a very mild glossy eye phenotype (Fig. S2B).

The neuronal distribution of the signal is confirmed by immunostaining with mAb24B10, which specifically recognises chaoptin, a GPI-linked cell surface glycoprotein that is present only on photoreceptor neurons and their axons (Fig. 1E) (Reinke et al., 1988; Zipursky et al., 1985). There is also intense 80S ribosome signal in enlarged foci at the tips of the R7 and R8 axons in the medulla region (Fig. 1E), which is probably in growth cones (Prokop and Meinertzhagen, 2006). Strong signals in photoreceptor growth cones are also apparent during pupal development (Fig. S2A). By comparing the pattern of the 80S signal with that of chaoptin, which mostly stains the periphery of the growth cones (compare insets in Fig. 1E), it is clear that the most intense ribosome signal is inside the growth cones. Comparison of the 80S signal with that of mCD8-GFP, another plasma membrane marker (Lee and Luo, 1999), which is evenly distributed along the axon (Fig. S1B, panel I versus panel II), also supports the conclusion that the whole interior of the growth cones must be replete with 80S ribosomes.

**Fig. 2.**
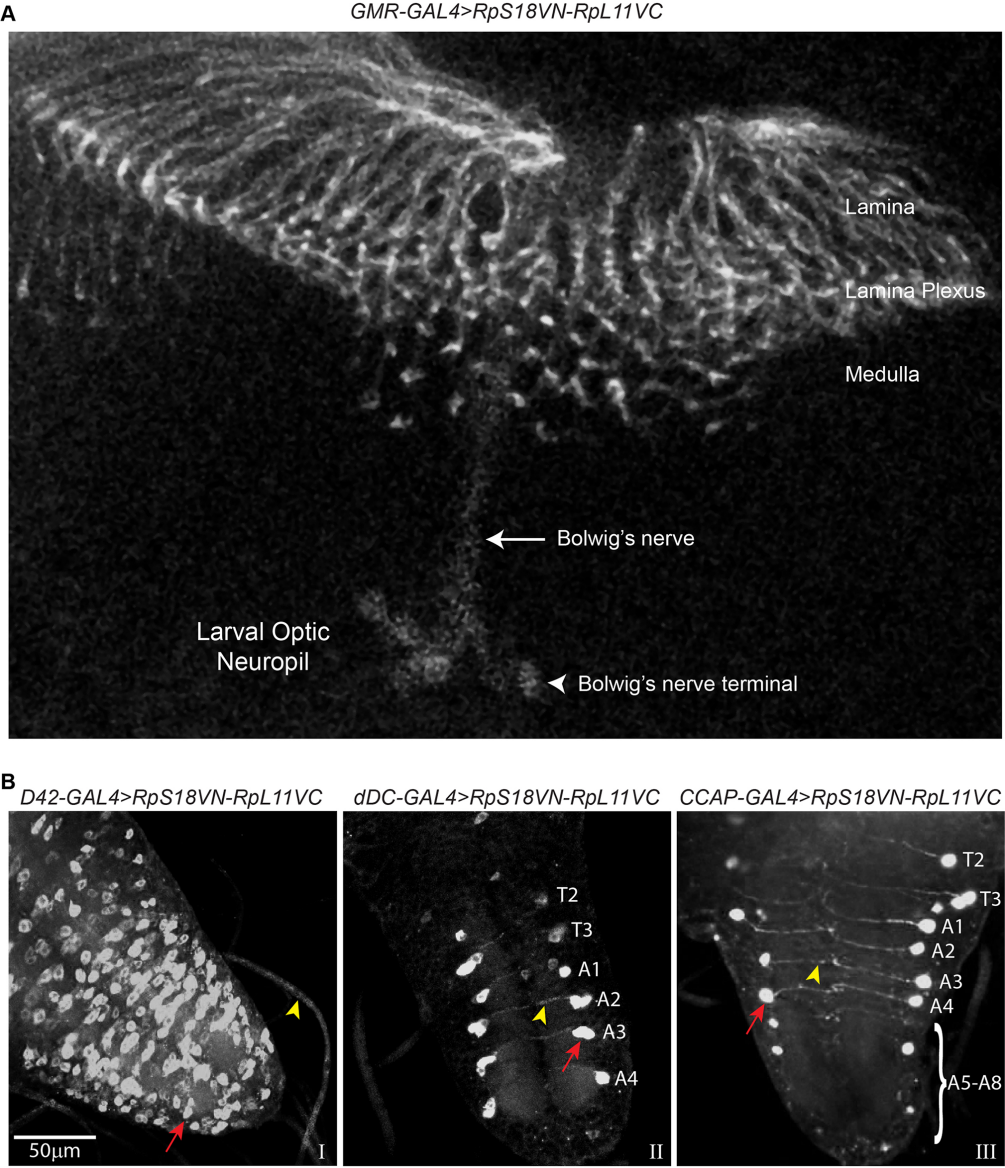
**Visualisation of Ribo-BiFC signals in mature axons.** (A) Distribution of the RpS18VN-RpL11VC reporter signals (grey) in the developing photoreceptors axons in one of the larval brain’s optical lobes and in mature axons of the Bolwig's nerve (arrow), as well as at the Bolwig's nerve terminals in the larval optic neuropil (arrowhead). (B) Visualisation of the Ribo-BiFC signal in specific mature neurons of different thoracic (T 2-3) and abdominal (A 1-8) segments of the larval ventral nerve cord demarcated by the expression of *D42-GAL4* (panel I), *dDC-GAL4* (panel II) and *CCAP-GAL4* (panel III). Yellow arrowheads indicate some of the neuronal projections and red arrows indicate cell bodies of some individual neurons in the ventral nerve cord.

We also found signals in the axons of functional adult fly photoreceptors (Fig. S2A). Although the Ribo-BiFC signal is weaker than in developing photoreceptor axons, the reduction is probably a consequence of reduced expression of the *GMR-GAL4* expression in adult flies, as this is also apparent when expressing mCD8-GFP alone (unpublished data). To test further whether ribosomes are present in the axons of mature neurons, we examined the Bolwig's organ. This is the organ of sight/light-sensation of the larva. It consists of a bilateral bundle of 12 photoreceptors near the mouth-hook at the anterior of the animal, which projects their axons in a nerve that joins with the optical stalk of the eye-disc before entering the brain optic lobe and terminates in a distinctive small region of the medulla representing the larval optical neuropil in each brain hemisphere (Fig. 1C) (Hofbauer and Campos-Ortega, 1990; Larderet et al., 2017). Within the neuropil, synapses are formed with the lateral neurons required for the circadian behaviour of the larva as well as the other neurons comprising the larval optical system (Helfrich-Förster et al., 2002; Keene et al., 2011; Larderet et al., 2017). We detected clear Ribo-BiFC signals along the Bolwig's nerve and at its terminals in larval optical neuropil (Fig. 2A).

We also examined the distribution of 80S ribosomes in other types of neurons by expressing the reporters using different GAL4 drivers (see Materials and Methods): *D42-GAL4* is expressed in motor neurons (Fig. 2B, panel I); and *DdC-GAL4* and *CCAP-GAL4* drive expression in pairs of laterally located neurons that are present in each segment of the brain ventral nerve cord, the axons/dendrites of which project to the midline (Fig. 2B, panel II and III, respectively). As in photoreceptor neurons, the Ribo-BiFC signals from 80S ribosomes are brighter in the cell bodies, but are apparent along the full length of the axons.

### Ribosomes in the distal regions of photoreceptor axons incorporate less puromycin

The classic way to assay for translation is to monitor ribosome-catalysed incorporation of puromycin into the C-terminal of nascent peptides, either radiochemically (Nathans, 1964), or more recently by immunostaining (David et al., 2012; Schmidt et al., 2009). When we incubated salivary glands with puromycin briefly to minimise diffusion of puromycylated peptides away from translation sites, as previously discussed (McLeod et al., 2014), we saw a good correlation between the 80S BiFC and puromycin signals (Al-Jubran et al., 2013). Puromycin immunostaining has also been recently used to visualise local translation in growth cones of axons that project from *Xenopus* retinal ganglion cells (Cioni et al., 2019), and mouse brain synaptosomes (Hafner et al., 2019).

**Fig. 3.**
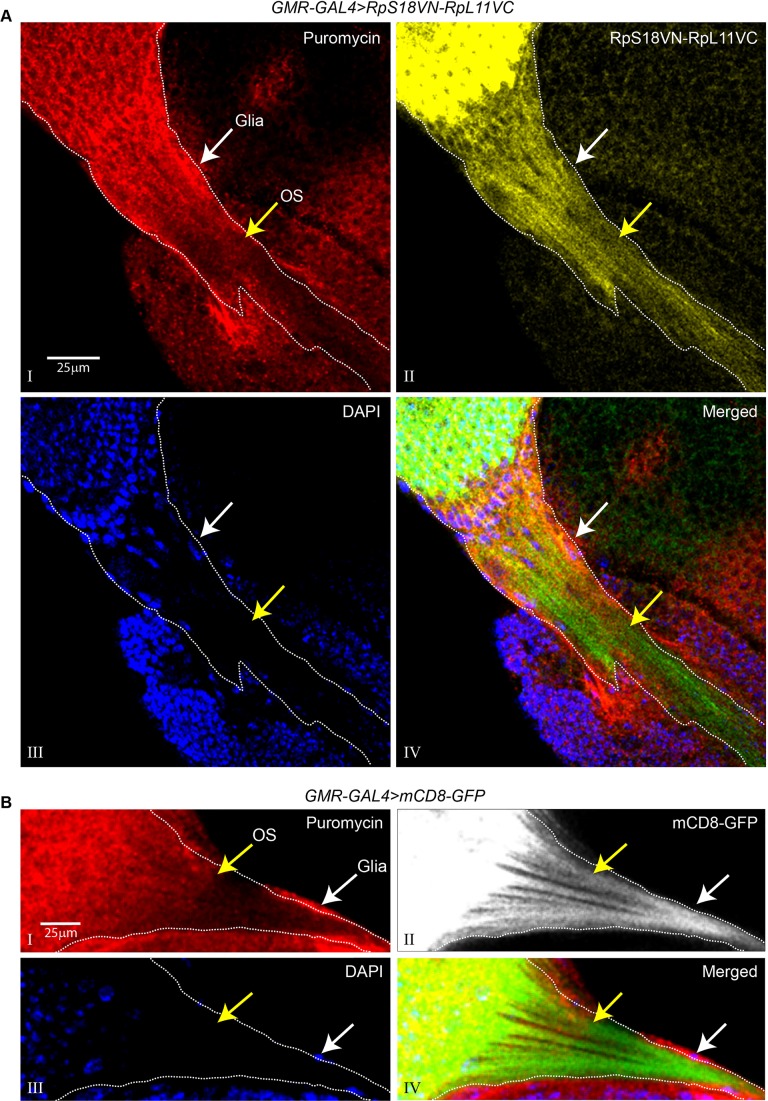
**Distal regions of growing photoreceptor axons incorporate relatively less puromycin.** (A) Immunocalisation of puromycin incorporation (red signal, panel I) in tissues expressing RpS18VN-RpL11VC in the photoreceptors via *GMR-GAL4* (yellow, panel II), DAPI staining (blue, panel III) shows the individual nuclei and highlights a monolayer of cells (white arrows), probably glia, surrounding the optic stalk (OS) (yellow arrow); the merged multicolour image highlights the overlap between the puromycylation and 80S signals in different regions of the photoreceptors (panel IV); the yellow arrow indicates the position of the optic stalk after which there is a reduced puromycylation signal compared to more proximal regions; the BiFC RpS18VN-RpL11VC signal is shown in green instead of yellow in the merged image for better contrast. (B) Immunocalisation of puromycin incorporation (red, panel I) in tissues expressing *GMR-GAL4* driven mCD8-GFP (grey, panel II), DAPI staining shows cell nuclei (blue, panel III); the merged image (panel IV) highlights the relatively more intense green colour in the distal segments of the optic stalk; and the mCD8-GFP signal is shown in green instead of grey for better contrast.

We took tissues in which the photoreceptors can be identified by expression either of Venus-based BiFC 80S reporters or tissue-targeted mCD8-GFP (Lee and Luo, 1999), labelled them and detected puromycylation by immunostaining. Inside the brain, the signal was weak and diffuse, and it could not be unambiguously traced to any of the photoreceptor projections or growth cones. However, a clearer pattern was apparent in the eye and optic stalk: it was most intense in the cell bodies in the developing retina and in the proximal regions of their axons (Fig. 3A shows distributions in a single longitudinal section of the optic stalk, and Fig. S3 shows projection images of multiple confocal sections of different preparations of the same tissue). Much of the distribution of the puromycylation signal is similar to that of 80S ribosomes (Fig. 3A, panel II and Fig. S3), but 80S ribosomes are only slightly less abundant in the distal parts of the axons that immunostained weakly for puromycin.

We considered whether the apparent proximal-to-distal gradient of the puromycin signal might be an experimental artefact caused by poor penetration of the antibody into the distal portions of the stalk that extends into the brain. To test this, we examined puromycin incorporation in detergent-permeabilised tissue, in which the photoreceptors were labelled by mCD8-GFP. The puromycin signal was again fainter in the distal regions of the permeabilised axons (Fig. 3B). Moreover, there was an intense puromycylation signal in the cells, possibly glia, that surround the entire length of the stalk, indicating that the antibody had free access (Fig. 3B, indicated by white arrows). The reduced incorporation of puromycin in the distal axonal regions seems therefore not to be caused mainly by a local shortage of ribosomes.

## DISCUSSION

Ribosome activation can be directly visualised by the fluorescence emitted as a result of the interaction between pairs of RPs in different subunits that: (a) are tagged with complementary parts of a BiFC-compatible fluorescent protein; and (b) are brought into close contact across the junction between subunits when a ribosome assembles. This technique, here named Ribo-BiFC, was previously used to visualise translating ribosomes in *Drosophila* S2 cells and salivary glands (Al-Jubran et al., 2013).

Although our previously described technique was not sensitive enough to visualise ribosomes in all neurons, here we described an improved version of this technique. Ribo-BiFC employs UAS-regulated transgenes that express pairs of neighbouring RPs (RpS18/RpL11 and RpS6/RpL24) tagged with BiFC-compatible complementary fragments of Venus fluorescent protein. These new transgenes allow a straightforward and sensitive visualisation of 80S ribosomes in *Drosophila* neurons and clearly detect assembled ribosomes in the axons and growth cones of developing photoreceptors, as well as in the axons of mature neurons, including larval photoreceptors. Here ribosome signals are also detected at the terminals located in the optical neuropil where synapses are formed with other neurons of the larval visual circuit (Larderet et al., 2017). We predict that the sensitivity of this method could be further increased by genetically combining multiple copies of the transgenes we generated (several P-element inserts are available; see Materials and Methods). These, together with the previously described UAS transgenes encoding individual GFP or RFP-tagged RPs, should provide useful tools to distinguish between inactive ribosomal subunits and assembled and actively translating ribosomes in *Drosophila* (Rugjee et al., 2013). As the Venus BiFC complex is very stable and possibly the key determinant of the Ribo-BiFC high sensitivity, it is not suitable for monitoring rapid changes in translation (Al-Jubran et al., 2013). However, we propose that our Ribo-BiFC technique provides a method to visualise changes in the subcellular distribution of ribosomes during different stages of *Drosophila* development and physiological states that is technically more straightforward than others recently developed (Lee et al., 2016). We detected a correlation between the presence of assembled ribosomes and puromycin incorporation, but some of the ribosomes in distal regions of axons seemed not to incorporate puromycin. These may correspond to ribosomes that are either paused on mRNAs after translation initiation or have significantly lower elongation rates. Ribosome pausing has been proposed to be an evolutionarily conserved mechanism to regulate protein synthesis (Darnell et al., 2018). Perhaps a similar regulatory mechanism operates on ribosome-loaded mRNAs present in axons of photoreceptors that are still growing and not yet active in the larval stage (Mencarelli and Pichaud, 2015).

## MATERIALS AND METHODS

### Fly stocks

Generation of the transgenes expressing the YN and YC YFP BiFC fragments or simply GFP or RFP tagged ribosomal proteins (RPs) has been previously described (Al-Jubran et al., 2013; Rugjee et al., 2013). The constructs expressing the RPs tagged with either the VN (1–173) and VC (155–238) fragments were similarly generated, cloned in the pUAST vector (Brand and Perrimon, 1993), and transgenic flies produced by P element-mediated transformation of standard *yw* strain (Bestgene). The *Fkh-GAL4* transgene was used to drive expression in salivary glands (Henderson and Andrew, 2000), *GMR-GAL4* expresses in the differentiated cells of the developing and mature eye including photoreceptors (Freeman, 1996), *D42-GAL4* expresses in motor neurons (Vonhoff et al., 2013), *dDC-GAL4* and *CCAP-GAL4* express in different groups of neurons in brain ventral cord (Vömel and Wegener, 2008). The *UAS mCD8-GFP* transgene encodes a membrane tethered GFP fusion protein used to visualise cell boundaries (Lee and Luo, 1999).

### Puromycylation and immunostaining

The brain-eye disc tissues of third-instar larvae from mentioned genotypes were dissected in M3 media and incubated with 50 µg/ml puromycin in M3 media for 1–10 min. Tissues were briefly washed with M3 media and transferred in 4% formaldehyde for 10 min. Following washing with PBST (0.1% Triton X-100 in 1× PBS) three times, tissues were incubated in blocking solution for 1 h at room temperature followed by mouse anti-puromycin antibody (David et al., 2012) (5B12, 1:500) overnight at 4°C. The mouse anti-chaoptin antibody (mAb24B10, 1:200, DSHB) was used as a photoreceptor neuron specific marker (Zipursky et al., 1985). Tissues were washed with PBST three times and incubated with anti-mouse-Cy3 secondary antibody (1:200) for 2 h at room temperature. Following washing the tissues were counterstained with 1 μg/ml DAPI (4–6-diamidino-2-phenyl indole, Sigma-Aldrich) and mounted with PromoFluor Antifade Reagent (PromoKine).

### Microscopy

The immunostaining signals in tissues were initially examined under Nikon Eclipse Ti epifluorescence microscope, equipped with ORCA-R2 camera (Hamamatsu Photonics). High resolution images were acquired using a Leica TCS SP2-AOBS confocal microscope equipped with HCX PL APO 40×/1.30-OIL-CS2 objective. The eye images of adult flies were captured using Zeiss Stemi 2000 CS microscope equipped with Zeiss AxioCam ICc 1 camera. The images were analysed with either Nikon NIS Elements or Fiji (Schindelin et al., 2012) and figures were prepared using Adobe Illustrator.

## Supplementary Material

Supplementary information
