# Supplementary material for: Visualisation of ribosomes in Drosophila axons using Ribo-BiFC
Source: Biol Open. 2020 Jan 2;8(12):bio047233. doi: 10.1242/bio.047233 (PMC6955225; doi:10.1242/bio.047233)
Supplement: Supplementary information [file biolopen-8-047233-s1.pdf]

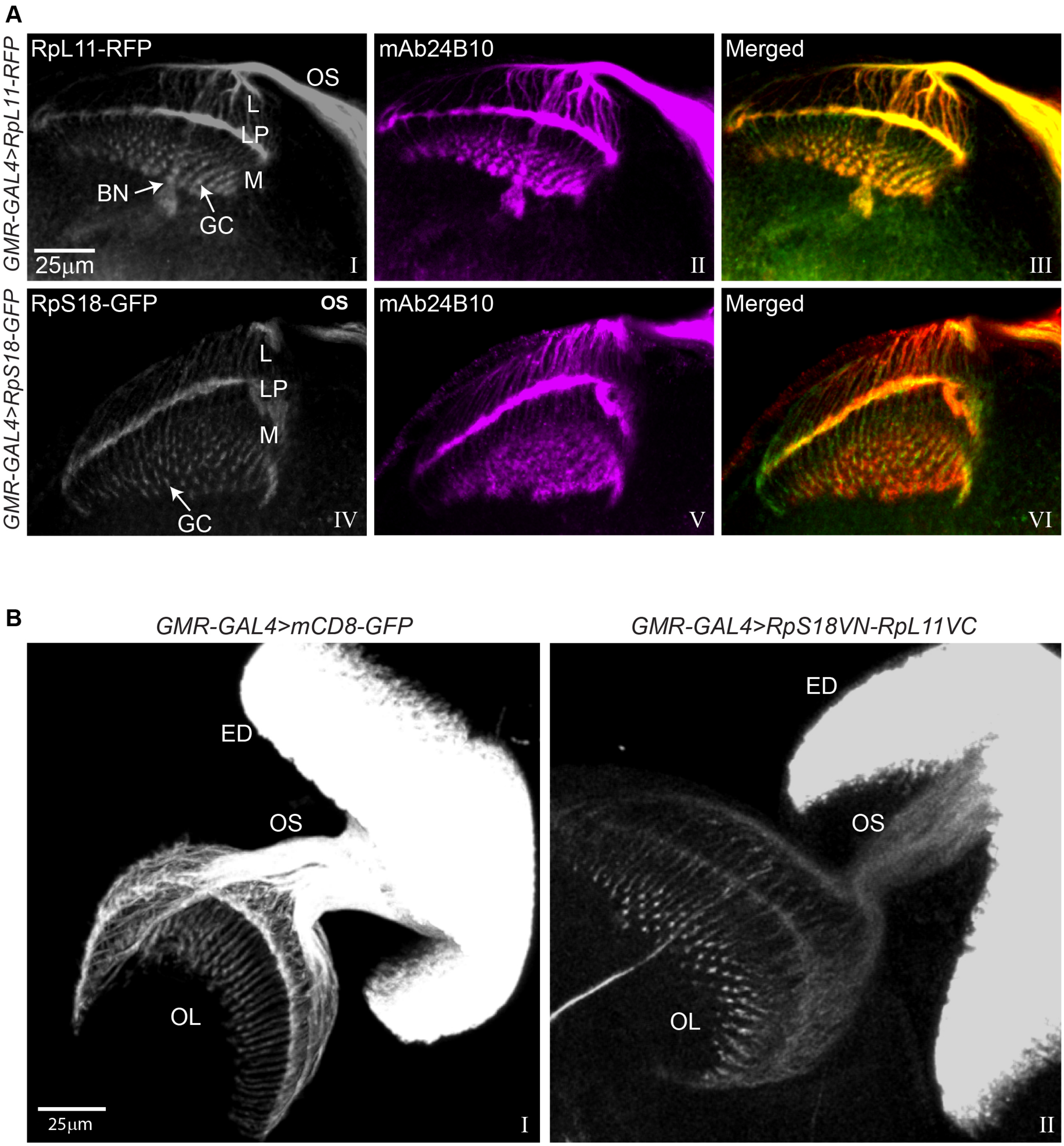
A
GMR-GAL4>RpL11-RFP
RpL11-RFP
OS
L
LP
M
BN
GC
25μm
I
mAb24B10
II
Merged
III
GMR-GAL4>RpS18-GFP
RpS18-GFP
OS
L
LP
M
GC
IV
mAb24B10
V
Merged
VI
B
GMR-GAL4>mCD8-GFP
ED
OS
OL
25μm
I
GMR-GAL4>RpS18VN-RpL11VC
ED
OS
OL
II

Fig. S1. The distribution of ribosomal proteins tagged with either GFP or RFP resembles that of Ribo-BiFC signals in photoreceptors. (**A**) Localisation of RpL11-RFP (green, panel I) or RpS18-GFP (green, panel IV) in the R1-R8 photoreceptors immunostained with mAb24B10 (magenta, panel II and panel V); the mAb24B10 is shown in red instead of magenta and both RpL11-RFP and RpS18-GFP in green in the corresponding merged images (panel III and panel VI) for better contrast. Labels refer to: OS- Optic Stalk; L- Lamina; LP- Lamina Plexus; M- Medulla, GC- Growth Cones, BN- Bolwig's Nerve. (**B**) Localization of *GMR-GAL4* driven mCD8-GFP (gray, panel I) and BiFC signal of the RpS18VN/RpL11VC reporter (yellow, panel II) in photoreceptors projected from eye disc (ED) via optic stalk (OS) to the optic lobe (OL).

Biology Open • Supplementary information

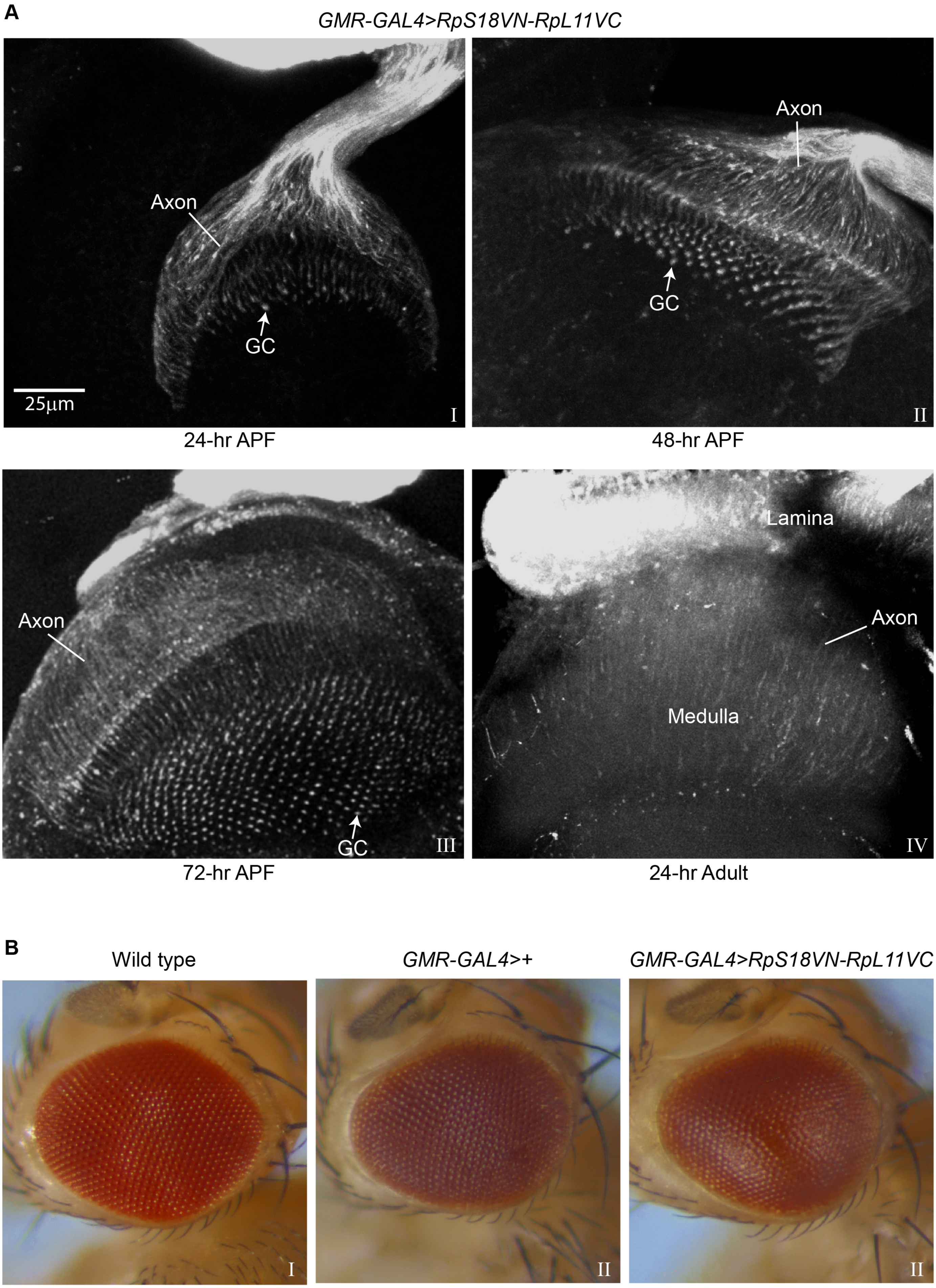
A
GMR-GAL4>RpS18VN-RpL11VC
Axon
GC
25μm
I
24-hr APF
Axon
GC
II
48-hr APF
Axon
GC
III
72-hr APF
Lamina
Axon
Medulla
IV
24-hr Adult
B
Wild type
GMR-GAL4>+
GMR-GAL4>RpS18VN-RpL11VC
I
II
II

Fig. S2. Ribo-BiFC signal in axons are detected at different stages of pupal development and in adult photoreceptors. Localization of Ribo-BiFC signal of RpS18VN/RpL11VC expressed via *GMR-GAL4* in developing photoreceptors at different pupal stages: 24-h (panel I), 48-h (panel II) and 72-h (panel III) after pupa formation (APF) and mature photoreceptors of 24-h old adult fly (panel IV). GC refers to Growth Cone (arrow). (B) Expression of the Ribo-BiFC reporter does not worsen eye development. Bright light micrographs of eyes of 24-h old adult flies: wild type (panel I), *GMR-GAL4>+* (panel II) and *GMR-GAL4>RpS18VN/RpL11VC* (panel III).

Biology Open • Supplementary information

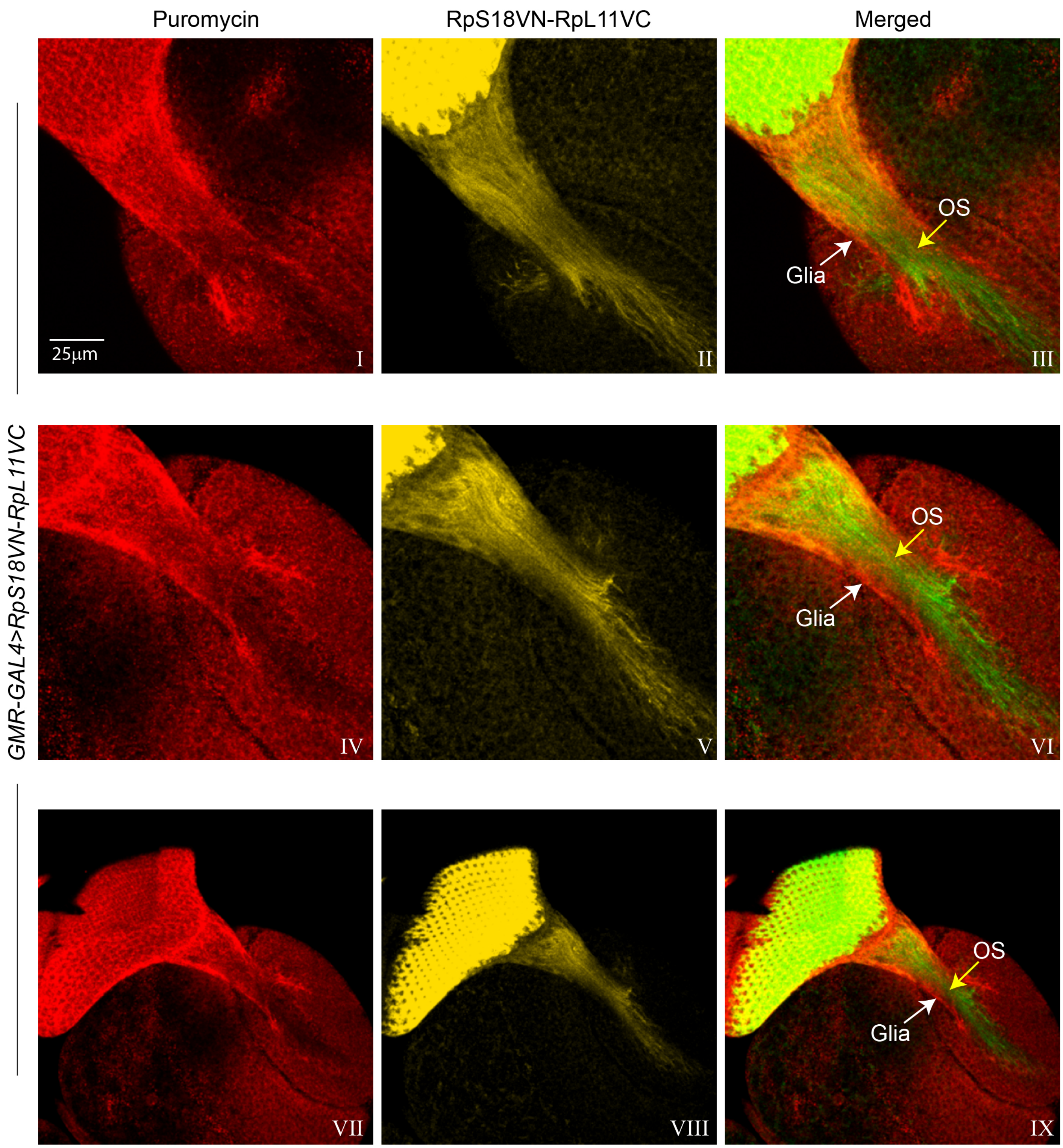

Biology Open • Supplementary information

Fig. S3. Distribution of puromycin incorporation and Ribo-BiFC signals in the developing retina and optic stalk of three different larvae. Projection images of the puromycin immunostaining signals (red, panels I, IV, VII) in tissues expressing *GMR-GAL4* driven RpS18VN-RpL11VC reporter in photoreceptors; the Ribo-BiFC signal is shown in yellow (panels II, V, VIII). The Ribo-BiFC signal is shown in green for better contrast in the merged image (panels III, VI, IX); the brighter green signal highlights the reduced puromycin incorporation in the distal segment of the optic stalk (OS) in all three preparations. White arrows indicate a layer of cells, probably glia, surrounding the optic stalks.
